# Layer-Wise Relevance Propagation Approach for Diagnosis of Drug-Naïve Men With Major Depressive Disorder Using Resting-State Electroencephalography

**DOI:** 10.1155/da/5512539

**Published:** 2025-09-23

**Authors:** Eun-Gyoung Yi, Miseon Shim, Seung-Hwan Lee, Han-Jeong Hwang

**Affiliations:** ^1^Department of Electronics and Information Engineering, Korea University, Sejong-si, Republic of Korea; ^2^Artificial Intelligence Smart Convergence Technology, Korea University, Sejong-si, Republic of Korea; ^3^Clinical Emotion and Cognition Research Laboratory, Department of Psychiatry, Inje University, Goyang-si, Republic of Korea; ^4^Department of Artificial Intelligence, Tech University of Korea, Siheung-si, Republic of Korea; ^5^Psychiatry Department, Ilsan Paik Hospital, Inje University, Goyang-si, Republic of Korea

**Keywords:** channel reduction, diagnostic tool, explainable artificial intelligence (XAI), major depressive disorders, resting-state EEG

## Abstract

The advancement of artificial intelligence (AI) tools utilizing electroencephalography (EEG) for diagnosing major depressive disorder (MDD) has shown significant progress. However, the practical implementation of these tools is often impeded by the large amount of EEG data required for training AI models and the lack of explanations for the MDD diagnoses. This study aims to develop an interpretable deep-learning-based computer-aided diagnostic system for diagnosing male MDD patients using explainable AI (XAI) algorithms. The CAD system was designed to facilitate the diagnostic process by using a reduced number of EEG channels and data length while enhancing understanding of the neurophysiological characteristics of male MDD. Resting-state EEG data were collected from 40 male MDD patients (20–63 years) and 41 gender-matched healthy controls (HCs, 19–61 years). A shallow convolutional neural network (CNN; Shallow ConvNet) model was utilized to distinguish between MDD patients and HCs. Relevance scores were extracted by the layer-wise relevance propagation (LRP) method, integrated with the Shallow ConvNet, to interpret the outcomes of the deep-learning-based CAD system. Additionally, changes in diagnostic performance were assessed by progressively reducing the number of channels using an LRP-based channel selection method, as well as EEG data length. Our XAI-based CAD system showed a high diagnostic performance of 100% when using the whole 62 channels with 180-s EEG data. A relatively high diagnostic performance over 90% was retained with only five channels with 60-s EEG data. Neurophysiologically meaningful brain areas, such as fronto-central, centro-parietal, and occipital areas, also revealed significant differences in relevance scores extracted by the LRP-method between the two groups. This study successfully developed a high performance and practical XAI-based CAD system for male MDD patients. Our developed CAD system not only achieves high diagnostic accuracy but also provides meaningful neurophysiological biomarkers for male MDD patients.

## 1. Introduction

Artificial intelligence (AI)-based computer-aided diagnosis (CAD) systems for major depressive disorder (MDD) using electroencephalography (EEG) have made significant advancements, with their performance considered suitable for clinical applications [1–7]. MDD is a prominent psychiatric condition exhibiting distinct EEG-based neurophysiological characteristics influenced by demographic factors, particularly gender [8–11]. For example, male MDD patients have shown different brain patterns in terms of both frequency power and functional connections as compared to female MDD patients [12]. Previous studies suggest that neglecting gender when analyzing EEG data could result in the development of CAD systems biased towards diagnoses for a specific gender [13, 14]. However, previous CAD studies have overlooked the distinctive neurophysiological characteristics of MDD patients associated with demographic information [15]. Therefore, in our previous study, we developed a gender-specific EEG-based CAD system for female MDD patients, thereby mitigating potential biases in diagnostic errors related to the opposite gender [16]. As our previous study focused only on female MDD patients, additional research specifically targeting male MDD patients is required. However, due to the lower incidence of MDD in males and the subsequent reduced research interest compared to female patients, an AI-based CAD system exclusively for male MDD patients has not yet been developed [17, 18].

Current advancements in EEG-based CAD systems indicate a shift towards utilizing deep-learning algorithms instead of using traditional machine-learning algorithms due to their enhanced performance [19, 20]. However, there is a lack of clear explanation for the superior performance of deep-learning-based CAD systems over traditional machine-learning-based CAD systems, leading to low reliability in the deep-learning-based CAD system. Moreover, from a psychiatric perspective, understanding diagnostic outcomes can not only support the reliability of diagnostic results but also provide insights into neurophysiological traits; therefore, interpreting the outcomes of the deep-learning-based CAD system is necessary [21–23].

Explainable AI (XAI) methods are key to understanding how deep learning models process input data to make diagnoses [24–26]. Notably, layer-wise relevance propagation (LRP), a prominent XAI approach, has been widely utilized in EEG data analysis [27, 28]. LRP can enhance an understanding of the decision-making process within a deep learning model by providing relevance scores derived from the decomposition of the model's output for each electrode. For instance, Charles et al. [29] have developed an LRP-based explainable CAD system for MDD patients that can simultaneously provide diagnostic results and neurophysiological insights through EEG spatio-spectral interactions. This system quantified reduced delta frequency characteristics of MDD patients based on relevance scores. However, with a diagnostic accuracy of 76.11%, which is generally lower than those of nonexplainable CAD systems, further improvements in diagnostic accuracy are essential [29].

Moreover, LRP can enhance the practicality of EEG-based CAD systems. The performance of deep-learning-based CAD systems is closely linked to the use of a larger amount of EEG data (channels) due to richer input data. However, increasing the number of EEG channels leads to higher costs for the recording system and requires more time to train deep-learning-based CAD systems, which potentially restricting their practical use [30, 31]. Therefore, to increase practicality, a reduction in the number of EEG channels is necessary, and an LRP-based channel selection method, prioritizing channels with the highest relevance scores, can be a viable solution.

The aim of this study was to develop an efficient and practical AI-based diagnostic assistant tool for male MDD patients using resting-state EEG data. To achieve this goal, we evaluated the diagnostic performance using a convolutional neural network (CNN) model with resting-state EEG data. We also investigated the distinctive neurophysiological traits of male MDD patients compared to healthy controls (HCs) using relevance scores extracted by the LRP method. Furthermore, we assessed changes in diagnostic performance with a decreasing number of EEG channels selected by the LRP-based channel-selection method, as well as with decreasing data length. To the best of our knowledge, this study is the first attempt to develop an efficient and practical XAI-based CAD system specifically designed for drug-naïve male patients with MDD, utilizing resting-state EEG data to simultaneously provide both high diagnostic performance and a comprehensive neurophysiological insight.

## 2. Materials and Methods

### 2.1. Participants

In the present study, we recruited 40 drug-naïve male MDD patients and 41 sex-matched HCs. Extending our previous work on a female-specific CAD system [16], we aim to develop a corresponding male-specific CAD system. The diagnosis of patients by a board-certified psychiatrist was based on the Diagnostic and Statistical Manual of Mental Disorders, 5th Edition (DSM-5) Axis I Psychiatric Disorders. The Hamilton Depression Rating Scale (HAM-D) and Hamilton Anxiety Rating Scale (HAM-A) were used to assess depression (mild: ≤13, *n* = 2; moderate: 14–18, *n* = 1; severe: 19–22, *n* = 9; very severe: ≥23, *n* = 28) [32] and anxiety (mild: ≤17, *n* = 1; moderate: 18–24, *n* = 8; severe: ≥25, *n* = 31) [33], respectively. These were used as an assistive diagnostic tool when clinical experts diagnosed the patients. Demographic data are summarized in Table 1. Patients were excluded if they met the following criteria: (1) abnormalities of the central nervous system, (2) medical histories of alcohol or drug abuse, (3) developmental delay, (4) history of head injuries with loss of consciousness and experience with electrical therapy (e.g., electroconvulsive therapy), and (5) psychotic symptoms lasting for at least 24 h. The HCs were recruited from the local community through local newspapers and posters. Individuals without any psychiatric medical history were recruited as HCs. This study was approved by the Institutional Review Board of Inje University Ilsan Paik Hospital [2015-04-316/2016-08-017] and conducted in accordance with The Code of Ethics of the World Medical Association (Declaration of Helsinki). All participants submitted written informed consent before the experiment.

### 2.2. EEG Recording and Preprocessing

Eyes-closed resting-state EEG data were recorded for 3–5 min at a sampling rate of 1000 Hz using a NeuroScan SynAmps2 (Compumedics USA El Paso, TX, USA) from 64 Ag/AgCl scalp electrodes evenly mounted on a QuikCap according to the extended 10−20 international system. The EEG data from 62 electrodes were analyzed, excluding the two reference electrodes (M1 and M2). The EEG data were bandpass filtered between 1 and 55 Hz. Subsequently, independent component analysis (ICA) [34] was employed to remove external artifacts (e.g., eye blinks, electrocardiogram, electromyogram, etc.), followed by the common average reference (CAR). The artifact-free EEG data were downsampled to 200 Hz to reduce computational demands and segmented into approximately 3-min intervals to ensure consistent lengths for all participants. MATLAB R2022a (MathWorks, USA) was used as the data processing tool.

### 2.3. XAI-Based Classification and Neurophysiological Interpretation

**Figure 1:**
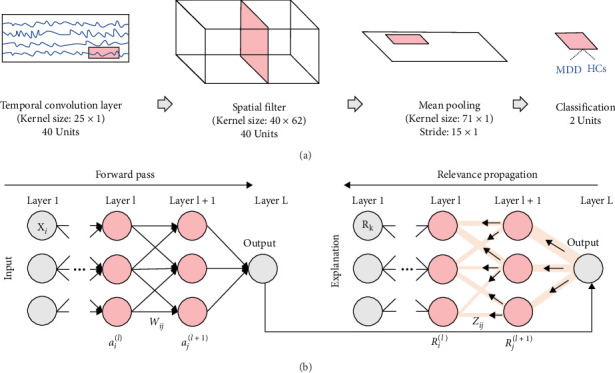
Scheme of (A) our deep learning-based CAD system and (B) layer-wise relevance propagation.

A Shallow CNN (Shallow ConvNet) model was employed to differentiate between MDD patients and HCs. The Shallow ConvNet was inspired by the Filter Bank Common Spatial Patterns (FBCSPs) and is specifically designed to decode spatiotemporal frequency power features in EEG data analysis [35]. The Shallow ConvNet architecture includes two different types of layers: two convolutional layers and one mean pooling layer, with a rectified linear unit (ReLU) used as an activation function (Figure 1A). Two convolutional layers were designed to capture temporal and spatial information for EEG frequency powers, respectively. The learning rate and dropout rate were set to 0.001 and 0.5, respectively. Moreover, a batch size of 2 and 200 epochs were set. When differentiating between two groups, a leave-one-out cross-validation (LOOCV) was used, where one subject was used for test data and the remaining subjects (*N −* 1) were used for constructing a classification model [36–40]. The *N −* 1 subjects were further divided into a training set and a validation set at a ratio of 4:1 to optimize the classification model.

The LRP method, one of the XAI algorithms, was used to explain the diagnostic results of our developed CAD system. LRP can quantify the contribution degree within the hidden layers, allowing for the reverse estimation of the features extracted by a specific hidden layer (Figure 1B) [27]. We computed the contribution score of the classification model (also known as the relevance score) of each hidden layer in a reverse manner using LRP on interpret the diagnostic results of our developed CAD system from neurophysiological point of view.

### 2.4. Reduction in Channel and Data Length

**Figure 2:**
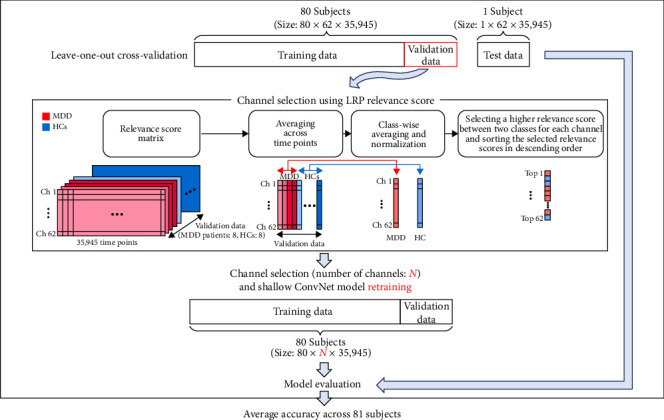
Scheme of the relevance score-based channel selection method. The channel selection process begins with training a model using EEG data from 64 subjects, followed by validating this model with data from a distinct group of 16 subjects. After the validation phase, relevance scores are calculated for each trial (subject) using the validation data. These relevance scores are then averaged across all time points, resulting in a 62 × 16 matrix (channels × number of validation data). The resultant matrix undergoes a process of class-wise averaging and normalization, culminating in a 62 × 2 matrix (channels × number of classes). The relevance scores for two classes (MDD patients and HCs) were compared for each channel and then the relevance score corresponding to the class with a higher relevance score was selected for the respective channel. The relevance scores for all channels were sorted in descending order and channels with higher relevance scores were sequentially selected.

The LRP method was adopted as a channel selection approach, as proposed by Nagarajan et al. [41].  Figure 2 shows the process of the relevance score-based automatic channel selection algorithm. LRP was applied to the validation set to generate a relevance score matrix (channel × time point), which was then averaged over time points and normalized for each class (MDD patients and HCs). The relevance scores for MDD patients and HCs were compared for each channel and then a higher relevance score was selected for the respective channel. The relevance scores for all channels were sorted in descending order and channels with higher relevance scores were sequentially selected to automatically reduce the number of EEG channels. Subsequently, the Shallow ConvNet model was retrained using data from the selected channels and was evaluated on the test dataset. The diagnostic performance of the CAD system was evaluated by 10 different numbers of EEG channels (1, 2, 3, 4, 5, 10, 15, 20, 25, and 62).

Additionally, we evaluated the impact of data length on the diagnostic performances of our developed CAD system. To this end, EEG data were segmented into eigh different lengths (10, 20, 30, 60, 90, 120, 150, and 180 s), and classification performances of each data length were evaluated for the 10 different numbers of channels (1, 2, 3, 4, 5, 10, 15, 20, 25, and 62). In total, 80 data combinations were analyzed to compute classification accuracy.

### 2.5. Statistical Analysis

We investigated the differences in classification accuracies across all combinations of various number of channels and data lengths using independent *t*-test with false rate discovery (FDR) correction. Moreover, we attempted to investigate the neurophysiological characteristics using relevance scores extracted by the LRP method. To this end, we normalized relevance scores for each subject and employed the independent *t*-test to identify significant differences in relevance scores between MDD patients and HCs.

## 3. Results

### 3.1. Classification Results

**Figure 3:**
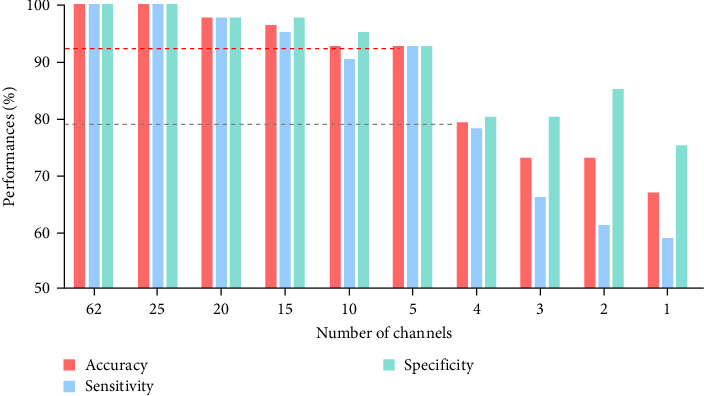
Diagnostic accuracy with respect to the number of selected channels. The red, blue, and green bars represent the accuracy, sensitivity, and specificity, respectively, in classifying MDD patients and HCs. The classification accuracy remained at 100% until the number of channels reduced to 25, and it was over 90% even with five channels.

The best classification accuracy, specificity, and sensitivity were all 100%, respectively, when differentiating male MDD patients and HCs using the EEG data of the whole 62-channels.  Figure 3 shows the changes in classification accuracies with respect to the number of EEG channels used for classification. Interestingly, although the number of EEG channels was reduced from 62 to 25, the classification accuracy remained at 100%. After using 20-channels, the classification accuracy was gradually reduced, but it remained over 90% even when using five channels. The classification accuracy dropped to less than 80% when using less than four channels. Detailed information about changes in classification performances are summarized in Table S1.

**Figure 4:**
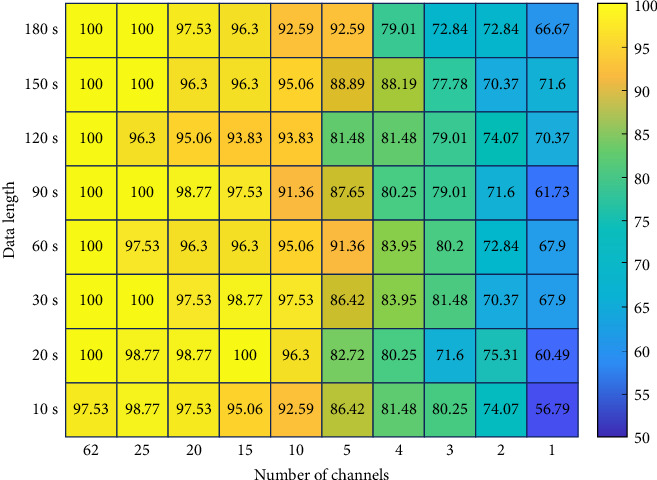
Classification accuracies across various combinations of numbers of channels and data lengths.

Figure 4 depicts the changes in diagnostic accuracies across the 80 combinations of data lengths and number of channels. We found that the diagnostic performances consistently remained above 90% even with the use of only five channels for 60 or 180-s EEG data, or when using 10 or more channels, irrespective of data length (FDR corrected *p* > 0.05).

### 3.2. Meaningful Brain Areas Based on LRP

**Figure 5:**
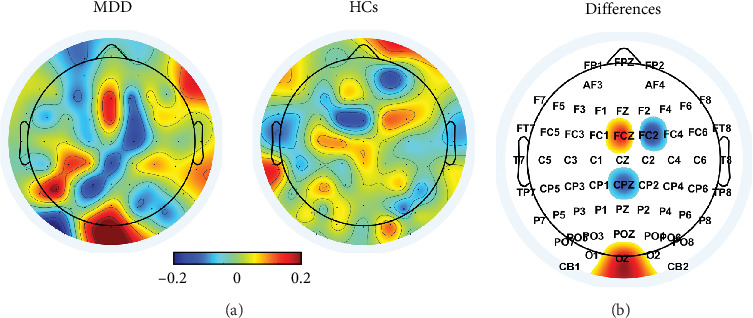
Topographical maps of (A) the channel-wise averaged relevance scores for each group and (B) statistical differences between two groups. In (A), red and blue colors indicate higher and lower relevance scores, respectively. In (B), red color indicates significantly higher relevance scores in male MDD patients compared to HCs, while blue color did significantly lower relevance scores.

Figure 5 shows the topographical maps of the channel-wise averaged relevance scores for male MDD patients and HCs when using the 62-channel EEG data. Male MDD patients showed relatively higher relevance scores in the fronto-central and occipital areas than those of HCs, whereas HCs did in the centro-parietal and right fronto-central regions. Male MDD patients showed the significantly higher relevance scores at FCz and Oz (FCz: *p*-value = 0.013 and Oz: *p*-value = 0.003) compared to HCs, but significantly lower at FC2 and CPz (FC2: *p*-value = 0.008 and CPz: *p*-value = 0.030).

### 3.3. EEG Channels Selected Based on LRP

**Figure 6:**
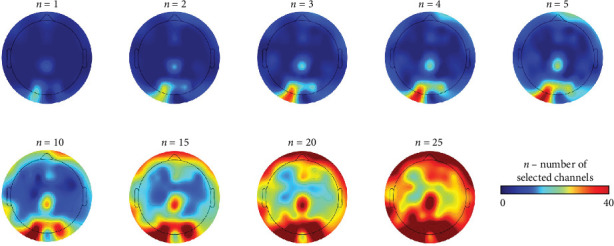
Topographical maps based on the frequency of selection for each EEG channel as the number of selected channels increases.

Figure 6 illustrates topographical maps based on the frequency of selection for each EEG channel as the number of selected channels increases (1, 2, 3, 4, 5, 10, 15, 20, and 25). When the number of selected EEG channels was increased from one to five, most selected channels were concentrated in the left parieto-occipital area. As the number of selected channels increased, the focus was expanded sequentially to include channels in the right parieto-occipital, central, and frontal areas. Upon reaching 25 selected channels, the distribution of EEG channels spanned across the entire brain.

## 4. Discussion

In this study, we developed a deep-learning-based CAD system using resting-state EEG data to assist diagnosis and understand the neurophysiological characteristics in male MDD patients. We also enhanced the practical feasibility of the CAD system by reducing EEG channels based on XAI method as well as data lengths. The highest diagnostic accuracy of 100% was achieved when using 62-channel EEG data, with diagnostic accuracy maintaining above 90% even when the number of selected channels was reduced to 5. Moreover, relevance scores extracted by our XAI-based CAD system showed significant differences between MDD patients and HCs in neurophysiologically meaningful brain areas, such as fronto-central, centro-parietal, and occipital areas.

Recently, deep-learning methods have been widely used to develop diagnostic tools instead of traditional machine-learning methods due to their efficiency, particularly in eliminating the need for hand-crafted feature extractions [42–44]. Indeed, an increasing number of studies have utilized deep-learning methods to develop CAD systems for MDD patients, demonstrating high diagnostic performance [45–47]. For example, Rafiei et al. [48], reported a diagnostic accuracy of 91.67% when differentiating MDD patients and HCs using 19-channel, 240-s resting-state EEG data based on a deep neural network (DNN) model. Moreover, Xu et al. [49], reported a higher diagnostic accuracy of 98.48% using 16-channel, 600-s resting-state EEG data utilizing a CNN-based model. While previous studies have shown high diagnostic performances in diagnosing MDD patients, no studies have investigated the practical feasibility of CAD systems in terms of the number of EEG channels and data length. In this study, we demonstrated that a relatively high diagnostic performance over 90% can be attained using only five EEG channels with either 60- or 180 s EEG data. Moreover, even with a data length of only 10 s, the classification performance was retained over 90% when utilizing 10 channels. The findings from this practicality investigation suggest that a high-performance CAD system can be developed using a limited number of EEG channels and short data lengths (e.g., five channels with 60-s EEG data or 10 channels with 10-s EEG data).

Abnormalities in neuronal activities have been reported mainly in frontal and central areas of male MDD patients based on resting-state EEG analysis. Male MDD patients showed higher alpha power in the frontal area and beta power in fronto-central areas than male HCs [50, 51]. Moreover, in terms of hemispheric analysis, male MDD patients exhibited significantly reduced neural activities in the left frontal area compared to male HCs [52]. Additionally, abnormalities in the occipital region were consistently observed statistically in general MDD patients, regardless of gender, compared to HCs [53–55]. However, to the best of our knowledge, no previous studies have provided the interpretation of the classification results of deep-learning-based CAD systems from a neurophysiological perspective. In the present study, significant differences in relevance scores between two groups were observed in fronto-central and parietal-occipital areas, regions associated with the characteristics of male MDD patients and general MDD patients, respectively. These results imply that our XAI-based CAD system effectively captured both the general and gender-specific neurophysiological characteristics of MDD patients, resulting in high diagnostic performance.

Interestingly, the channel locations that showed significant differences in relevance scores between the two groups closely matched those identified by the LRP-based channel selection method. When a limited number of channels (*n* ≤5) were selected, they were predominantly located in the central and occipital regions. This observation is consistent with previous studies reporting that patients with MDD exhibit abnormal frequency characteristics in the occipital regions compared to HCs [53, 56, 57]. The concentration of relevance score differences in these occipital areas suggests that our CAD system captures general neurophysiological abnormalities commonly observed in depression, reflecting distinct brain functioning patterns associated with MDD. As the number of selected channels increased, our CAD system progressively incorporated frontal regions, which resulted in further improvements in classification performance. Notably, male patients with MDD have been shown to exhibit distinct frontal characteristics, including abnormal frequency patterns and impaired functional connectivity, as compared to HCs [50, 58]. Therefore, the enhanced performance with more selected channels likely reflects the integration of both general features of depression (captured by occipital channels) and male-specific neurophysiological characteristics (captured by frontal channels). This underscores the biological plausibility of the LRP-based channel selection approach and its alignment with known MDD pathophysiology.

As the number of selected channels increased, the channels in the frontal areas began to be included. This indicates that the LRP-based channel selection method can effectively identify important channels related to the neurophysiological characteristics of MDD patients, thereby maintaining high diagnostic performance even with fewer channels. Moreover, compared to mainly using occipital channels (*n* ≤5) related to the neurophysiological characteristics of general MDD patients, diagnostic performance was improved when incorporating frontal channels related to male-specific characteristics. It indicates that gender-specific characteristics need to be considered along with the characteristics of general MDD to improve diagnostic performance when developing an EEG-based CAD system for diagnosing MDD patients.

Moreover, beyond physiological data-based CAD system, a wide range of alternative data sources has been utilized to develop AI-based diagnostic systems for depression, including social media data (e.g., video, audio, and text) and biological indicators (e.g., structural MR image and blood-based markers) [59, 60]. These studies have reported promising diagnostic performance, frequently achieving accuracies exceeding 90%. However, many of these approaches emphasize classification accuracy while offering limited insight into the underlying neurobiological or behavioral mechanisms. In contrast, our study integrates an XAI framework that not only yields high diagnostic performance but also visualizes and interprets the spatio-temporal patterns in EEG signals that are characteristic of MDD. This approach enables our CAD system to provide both robust predictive capabilities and interpretable neurophysiological insights, thereby enhancing its relevance for clinical application and translational psychiatry.

Despite the well-designed study, several limitations remain. First, we used a relatively small sample size of 81 subjects (40 drug-naïve male MDD patients and 41 male HCs), indicating the need for a larger database to improve the generalizability of our results. Second, our CAD systems was developed specifically for male MDD patients. To ensure broader applicability of our CAD system, an additional CAD system should be developed for female MDD patients. Eventually, an integrated CAD system that combines the two gender-specific CAD systems should be developed for use regardless of gender.

## 5. Conclusions

In this study, we successfully developed a practical resting-state EEG-based CAD system for male MDD patients using deep-learning technique, and investigated the diagnostic reasoning based on an XAI method. We obtained a high diagnostic accuracy of 100% using all EEG channels, and diagnostic performance remained above 90% even when the number of EEG channels was reduced from 62 to 5 through the LRP-based channel selection method. Additionally, significant differences in relevance scores were observed in neurophysiologically meaningful brain areas, such as the fronto-central, centro-parietal, and occipital regions, further validating the effectiveness of the LRP method in distinguishing between the two groups. Furthermore, as a future direction, we plan to explore more sophisticated diagnostic model architectures by modifying network layers—for instance, by incorporating deeper or hierarchical feature extraction blocks designed to capture higher-order, nonlinear, and cross-regional spatio-temporal interactions within EEG signals. Such advancements could help build a more robust system capable of maintaining high performance even with fewer EEG channels, thereby enhancing its clinical applicability.

## Figures and Tables

**Table 1:** Demographic data of patients with drug-naïve male major depressive disorder (MDD) and healthy controls (HCs).

	MDD	HC	*p*-Value
Cases (*N*)	40	41	—
Age (years)	33.88 ± 12.24	34.15 ± 11.37	0.9179
Education (years)	14.83 ± 1.06	15.27 ± 0.95	0.0507
HAM-D	24.58 ± 5.01	—	—
HAM-A	29.30 ± 6.67	—	—

*Note*: All participants are male. Statistical significance of age and education was evaluated using independent *t*-test.

Abbreviations: HAM-A, Hamilton Anxiety Rating Scale; HAM-D, Hamilton Depression Rating Scale.

## Data Availability

The data will be made available upon request.

## Supporting Information

Supporting Information Table S1. Detailed classification accuracies with respect to the number of channels (units: %).
