# Supplementary material for: Layer-Wise Relevance Propagation Approach for Diagnosis of Drug-Naïve Men With Major Depressive Disorder Using Resting-State Electroencephalography
Source: Depress Anxiety. 2025 Sep 23;2025:5512539. doi: 10.1155/da/5512539 (PMC12483754; doi:10.1155/da/5512539)

**<Supplementary material>**

Table S1. Detailed classification accuracies with respect to the number of channels (units: %)

|  | 62 | 25 | 20 | 15 | 10 | 5 | 4 | 3 | 2 | 1 |
| --- | --- | --- | --- | --- | --- | --- | --- | --- | --- | --- |
| Accuracy | 100.00 | 100.00 | 97.53 | 96.30 | 92.59 | 92.59 | 79.01 | 72.84 | 72.84 | 66.67 |
| Sensitivity | 100.00 | 100.00 | 97.56 | 95.12 | 90.24 | 92.68 | 78.05 | 68.85 | 60.98 | 58.54 |
| Specificity | 100.00 | 100.00 | 97.50 | 97.50 | 95.00 | 92.50 | 80.00 | 80.00 | 85.00 | 75.00 |

Table S2. EEG channels for different numbers of selected channels (ranked by selection frequency).

| Number of selected channels | Names of selected channels |
| --- | --- |
| 1 | O1 |
| 2 | O1, PO3 |
| 3 | O1, PO3, CB1 |
| 4 | O1, CB1, PO3, PO4 |
| 5 | O1, CB1, PO3, POZ, PO4 |
| 10 | O1, CB1, PO3, O2, PO4, CPZ, POZ, PO6, CB2, PO5 |
| 15 | O1, CB1, O2, PO4, PO3, PO6, POZ,  CPZ, CB2, PO7, PO5, PO8, TP7, CZ, FP2 |
| 20 | O1, PO4, O2, CB1, PO3, CB2, PO6, POZ, CPZ, PO7, FP2, PO5, TP7, PO8, FP1, FPZ, CZ, P3, F7, F8 |
| 25 | O1, PO4, O2, CB1, POZ, PO3, PO6, PO7, CPZ, CB2, FP2, PO8, P3, PO5, CZ, TP7, F7, FT8, FP1, F8, P5, FPZ, PZ, P4, TP8 |

Figure S1. Flowchart for the LRP-based channel selection process and data length variation experiments in the proposed EEG-based diagnostic system.


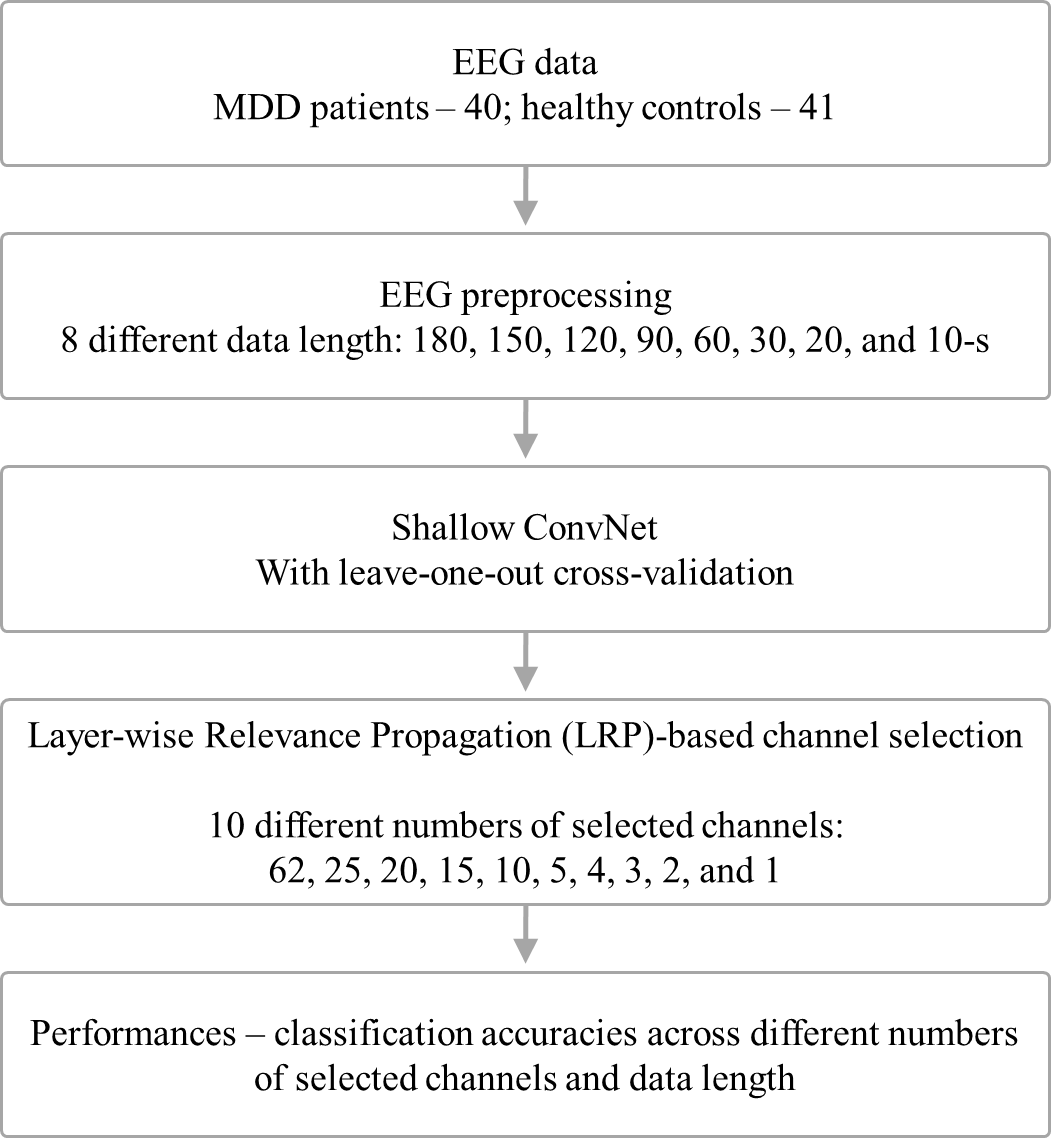

Supplement: Supporting Information — Table S1. Detailed classification accuracies with respect to the number of channels (units: %). [file 5512539.f1.docx]
